# A Reproducible Videogrammetric Methodology for Kinematic Reconstruction of Road Accidents from Uncalibrated Stationary Cameras with GUM-Based Uncertainty Evaluation

**DOI:** 10.3390/s26216868

**Published:** 2026-10-30

**Authors:** Miroslav L. Kokalarov, Ivan R. Georgiev, Ivaylo N. Nikolaev, Slavi G. Georgiev, Georgi P. Georgiev, Meglena D. Lazarova

**Affiliations:** 1 Department of Machine Elements, Materials Science and Chemistry, Todor Kableshkov University of Transport, 158 Geo Milev Str., 1574 Sofia, Bulgaria; mkokalarov@vtu.bg; 2 Department of Information Modeling, Institute of Mathematics and Informatics, Bulgarian Academy of Sciences, Acad. Georgi Bonchev Str., Block 8, 1113 Sofia, Bulgaria; irgeorgiev@math.bas.bg (I.R.G.); sggeorgiev@math.bas.bg (S.G.G.); 3 Department of Applied Mathematics and Statistics, University of Ruse “Angel Kanchev”, 8 Studentska Str., 7004 Ruse, Bulgaria; irgeorgiev@uni-ruse.bg (I.R.G.); sggeorgiev@uni-ruse.bg (S.G.G.); 4 Department of Heat, Hydraulics and Environmental Engineering, University of Ruse “Angel Kanchev”, 8 Studentska Str., 7004 Ruse, Bulgaria; nikolaev@uni-ruse.bg; 5 Department of Security and Safety, Faculty of Law, Varna Free University “Chernorizets Hrabar”, 84 Yanko Slavchev Str., 9007 Varna, Bulgaria; georgi.georgiev@vfu.bg; 6 Department of Mathematical Modelling and Numerical Methods, Faculty of Applied Mathematics and Informatics, Technical University of Sofia, 8 Kliment Ohridski Blvd., 1000 Sofia, Bulgaria

**Keywords:** videogrammetry, accident reconstruction, surveillance camera, camera as a sensor, homography, lens distortion, kinematic model, measurement uncertainty, Monte Carlo, stopping distance

## Abstract

Uncalibrated surveillance recordings can support road-accident reconstruction, but uncertain geometry, undocumented frame timing, and occlusion constrain the interpretation. This study presents a reproducible workflow combining geometric correction, feature tracking, kinematic regression, and conditional uncertainty propagation. An anonymised car–motorcycle collision illustrates the distinction between numerical reproducibility and independent measurement validation. Under a nominal 15 fps time base, the motorcycle two-phase fit gives an initial speed of 111.2 km/h with an expanded conditional uncertainty of 3.0 km/h (k = 2). The early visible car segment has a mean regression slope of 47.9 km/h; its later motion is fitted separately because a uniform initial phase is not established. Synthetic experiments assess estimator errors, interval coverage, geometric sensitivity, and changes in uncertainty-source ranking under specified conditions. The stopping analysis remains a scenario conditional on reaction, braking, timing, and geometric assumptions, with extrapolation outside the motorcycle observation interval. Missing independent calibration checks and frame timestamps prevent an end-to-end accuracy claim. The contribution is a transparent workflow separating observations, assumed distributions, model sensitivity and unresolved systematic effects.

## 1. Introduction

Video recordings from stationary surveillance (CCTV) cameras have become a primary source of objective information on the motion of vehicles in the seconds preceding a collision. Whereas trace-based reconstruction infers pre-impact motion from rest positions, tyre marks, and deformation [1,2], a video recording provides a direct, densely sampled position–time record of every visible road user. From the point of view of measurement, however, a surveillance camera is an unfavourable instrument: its interior orientation is unknown, its lens exhibits radial distortion, its frame rate is low and not necessarily constant, it observes the carriageway under strong perspective, and the tracked objects are intermittently occluded. Any quantitative use of such a recording is therefore a measurement with a sensor never intended for measurement, and the credibility of the result depends on whether the sensor’s metrological characteristics have been identified and their effect on the result quantified.

Speed estimation from video has an established literature. Doğan et al. [3] estimated vehicle speed from side-view images of an uncalibrated camera with an accuracy of about ±1 km/h against a GPS reference; Dahl and Javadi [4] derived the probability density of the speed estimate from the frame-rate statistics; Kim et al. [5] proposed a virtual-plane method validated against GPS and applied to forensic videos; Epstein and Westlake [6] and Bruehs and Stout [7] used reverse-projection photogrammetry with frame-timing metadata; Paolino and Zampa [8] evaluated an open-source tracking tool; Snyder et al. [9] fitted a speed profile to tracked positions by optimisation; Choi and Kong [10] addressed occluded scenes with cross-ratio constraints; Abramowski and Reński [11] reviewed quantitative analysis of stationary and on-board recordings and discussed an uncertainty estimate. In Bulgaria, Lyubenov et al. developed an expert system for the reconstruction of accidents [12] and studied the dynamic parameters of vehicles with GPS-based logging [13], including the effect of a low logging frequency on the estimated kinematic parameters [14]—a question that arises in an aggravated form with surveillance video. Roadside-camera calibration and monocular measurement have been treated in this journal by Li et al. [15], Sun et al. [16] and Lottefier et al. [17], the latter documenting the systematic errors of homography-based trajectory reconstruction. Uncertainty in accident reconstruction has been analysed with Monte Carlo methods since the 1990s [18,19] and formalised by Wach [20]; the recent UK Forensic Science Regulator notification on speed estimation from video footage explicitly requires an evaluation of uncertainty for every case [21].

The present work addresses a practical integration problem: how to document the complete chain from a partially characterised recording to kinematic estimates and their limitations. Geometric calibration, piecewise regression, and Monte Carlo uncertainty propagation [22,23] are established methods. Their combination is useful only when the assumptions and provenance of each stage are explicit. In particular, a small trajectory-fitting residual cannot by itself establish the accuracy of the image-to-road transformation, and a nominal frame rate cannot substitute for verified frame timing. These distinctions motivate the workflow and the critical assessment of its case-study application.

The contributions are an integrated protocol for stationary-camera reconstruction (Appendix A); an explicit connection between a road-plane homography and a one-dimensional projectivity; a continuous two-phase kinematic formulation with a profiled transition instant; and a documented framework for propagating assumed input uncertainties to speed, collision-related quantities and stopping scenarios. The transition estimator is a piecewise-regression procedure rather than a new class of estimator. The case study illustrates its use under restricted evidence, while synthetic experiments assess its behaviour under specified data-generating assumptions. The contribution is therefore methodological integration, reproducibility, and explicit treatment of limitations; we do not assume universal superiority over existing reconstruction methods.

The remainder of the paper is organised as follows. Section 2 describes the sensor model, the geometric transformations, the kinematic model, the uncertainty budget, its analytical verification, the numerical implementation, and the validation design. Section 3 reports the synthetic verification and the case study. Section 4 discusses sensitivity, applicability conditions, and limitations. Section 5 concludes.

## 2. Materials and Methods

### 2.1. The Surveillance Camera as a Measuring Sensor

The measuring chain begins with the recording file. Surveillance recorders may store H.264 streams in proprietary containers, such as the DAV container reported here, at a nominal frame rate that does not establish the decoded sampling times. Spatial sampling, frame timing, and lens geometry separately limit the measurement. Conversion of image-localisation error to road-coordinate error requires a position-dependent Jacobian for a verified camera mapping. Resolution and an approximate scene span alone do not establish the local metres-per-pixel scale. In particular, an illustrative feature width of several pixels [24] is not a measured standard uncertainty for this case. The metre-based residual scales adopted below are conditional noise assumptions, not independently characterised pixel errors [25,26,27,28,29].

The recommended time-base verification uses presentation timestamps of all decoded frames [30], obtained, for example, with FFprobe (-show_frames). For N retained frames with first and last timestamps t_1_ and t_N_, the average interval rate is (N − 1)/(t_N_ − t_1_). The complete interval sequence must also be inspected: an average rate alone does not reveal local jitter, dropped frames, or duplicates. A uniform time-scale error propagates proportionally into speed and quadratically into acceleration. In the present case, individual presentation timestamps were not available for this analysis. The nominal rate of 15 fps is consequently an assumption. The allowance of ±0.5% used in the original uncertainty calculation represents a sensitivity scenario for a global rate error, not an established bound on all timing errors. Dividing the frame counts by the nominal 18 min duration gives about 14.93 fps for the primary camera and 14.08 fps for the auxiliary camera (Table 1); these ratios cannot establish local sampling times because the duration is nominal.

### 2.2. Scene Geometry and Coordinate Systems

**Figure 1:**
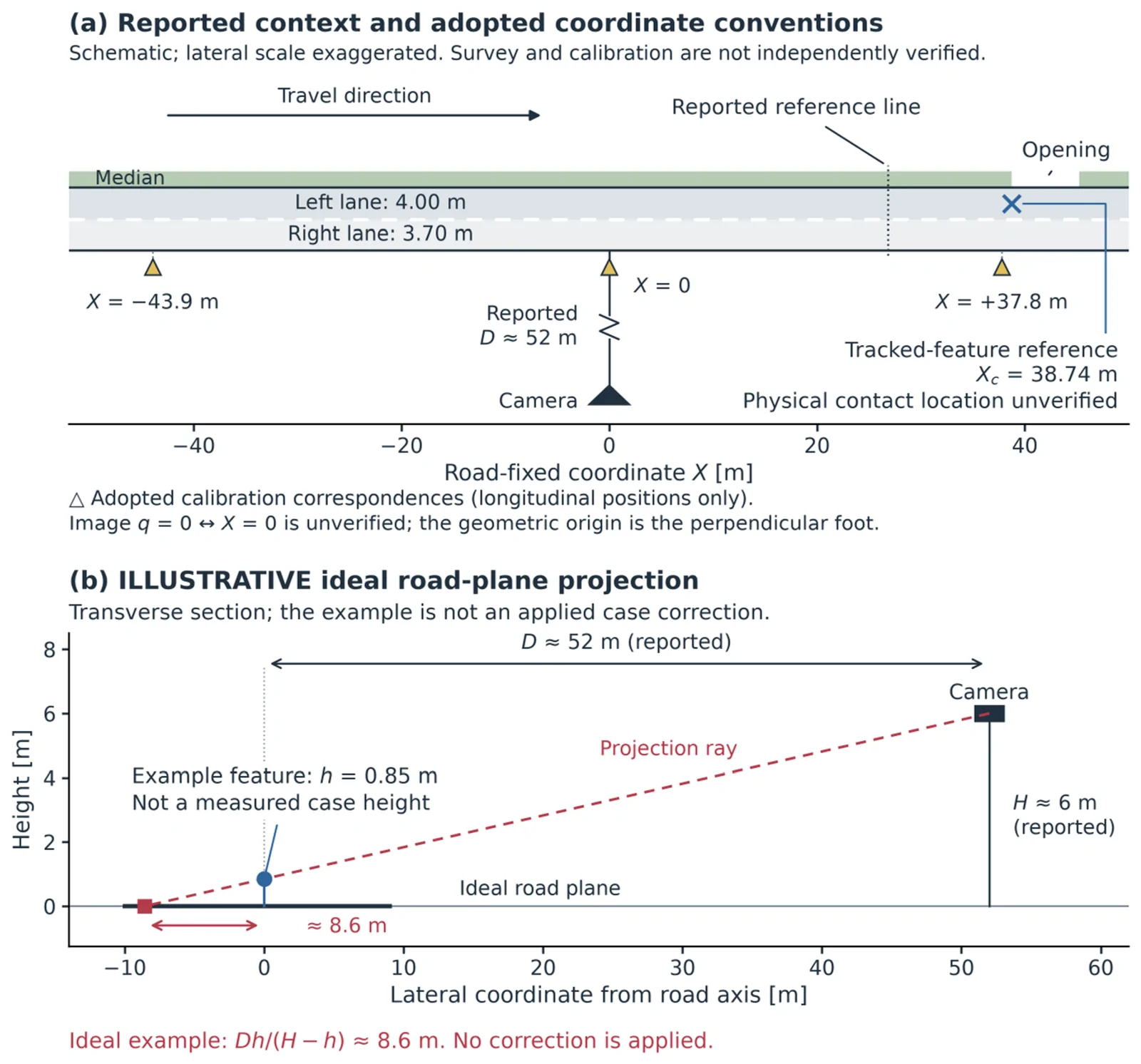
Reported observation geometry and coordinate conventions (schematic, anonymised): (**a**) plan view with road-fixed axis X, control-point locations, the reference Xc used in the calculation and the median opening; (**b**) transverse section illustrating camera height H, lateral distance D and the projection of a feature at height h above the road plane. The schematic does not establish the physical contact location or measure the feature-to-contact offset; Xc denotes the tracked-feature reference described in Section 2.2.

A road-fixed Cartesian system is defined in the road plane with the X-axis along the direction of travel and the origin at the foot of the perpendicular from the camera projection centre to the road axis (Figure 1). The reported scene geometry has lateral offset D ≈ 52 m and camera height H ≈ 6 m. The control-point coordinates originate from the scene survey; the rectangular ±0.20 m survey allowance used here is an assumed sensitivity range, not a documented tape resolution or measured survey uncertainty. The case calculation uses t = T − Tc and q = X − Xc, where Tc is the nominal time assigned to the reported contact frame and Xc is the mapped position of the tracked car feature in that frame. Xc is not an independently established physical contact point. A feature-to-contact offset would be needed to make that identification. Translation of the reference system alone does not change velocities or accelerations.

### 2.3. Lens-Distortion Model and Estimation

In the prospective workflow, image coordinates are referred to the principal point, and radial distortion is modelled using Brown-type coefficients [31,32]. With the correction-direction convention used here, corrected radius r′ is expressed in terms of observed radius r as

r′ = r (1 + k_1_ r^2^ + k_2_ r^4^), (1)

where neglecting tangential terms is a modelling choice that requires checking for the lens under study. If a calibration target is unavailable, coefficients k_1_ and k_2_ may be estimated from straight scene lines by minimising residual curvature, following the plumb-line principle [31], or using a documented distortion model in the tracking software [33]. Coefficients, the forward or correction convention, and residual straightness errors must be retained. When measured, residual errors can be propagated through the local image-to-road scale. Lens correction should precede projective transformation, but this processing order does not imply statistical independence of their errors. These are recommended calibration steps; their successful execution and residual accuracy are not established for the present case.

For the case study, the available documentation describes distortion compensation qualitatively but does not provide numerical coefficients or post-correction straightness residuals. Accordingly, this documentation does not support a verified distortion correction or a quantitative residual-distortion bound. Kinematic fit residuals measure agreement with the chosen motion model; systematic geometric errors can be absorbed into the fitted trajectory and therefore need not appear in those residuals. The numerical uncertainty budget must be interpreted conditionally on the adopted geometry, with residual lens distortion identified as an incompletely characterised source rather than assumed to be bounded by tracking noise.

### 2.4. Road-Plane Homography and Its One-Dimensional Reduction

For a stationary camera, points in the image and road planes are related by a planar homography [34]. Here H maps image coordinates to road coordinates: [X, Y, 1]^T^ is proportional to H[u, v, 1]^T^, with eight independent matrix parameters. In this direction, the inhomogeneous equations are:

X = (h_11_ u + h_12_ v + h_13_)/(h_31_ u + h_32_ v + 1),   Y = (h_21_ u + h_22_ v + h_23_)/(h_31_ u + h_32_ v + 1), (2)

which give the eight-parameter perspective transformation [35]. In a prospective calibration, H can be estimated from at least four non-collinear correspondences using a normalised direct linear transformation, with robust outlier handling where sufficient redundant controls are available [34,36]. Residuals at determining controls assess fit, while independent check points are needed to assess transformation accuracy. No such fully independently checked homography is claimed for the present case.

When the tracked point moves along a straight line in the road plane (a lane axis), its image moves along a straight line in the image plane, and the correspondence between an image abscissa q measured along that line and the road abscissa X is a one-dimensional projectivity,

X(q) = (a q + b)/(c q + 1),(3)

which is a line restriction of a planar projective mapping and is determined by three-point correspondences. Setting b = 0 imposes the correspondence q = 0 ↔ X = 0; this is an additional calibration assumption, not information supplied merely by renaming the origin. With that correspondence imposed, two further control points determine a and c exactly. More controls permit a redundant fit and independent checks. The reduction requires a suitable tracked-feature locus and a consistent geometric convention. The actual feature height and deviations from the calibrated line must be assessed before applying it to a case. For illustration, the simple estimate ΔY·X/D gives about 0.8 m for ΔY = 1 m, X = 40 m and D = 52 m; this is an assumed geometric example, not a measured case error, and it is not included in the principal uncertainty budget. If the line assumptions fail, a full road-plane mapping requires suitable two-dimensional calibration and tracking, with a separate treatment of off-plane features.

For Equation (3), the local scale is dX/dq = (a − bc)/(cq + 1)^2^, reducing to a/(cq + 1)^2^ when b = 0. Calibration sensitivity is position-dependent and may increase outside the interval spanned by the control points. A numerical scale range must therefore be evaluated over the actual observed coordinate interval, with the coordinate units stated explicitly. Exact agreement with the points that determine a projectivity is not an independent validation of the transformation.

### 2.5. Tracked Points Above the Road Plane

A road-plane homography applies to points on that plane. For an ideal pinhole camera with projection centre C = (0, −D, H) and a feature P = (X, Y, h), the ray through P intersects the road plane at Q with QX = HX/(H − h) and QY = −D + H(Y + D)/(H − h). Thus QX − X = hX/(H − h), while QY − Y = h(Y + D)/(H − h). For a feature on the reference lane (Y = 0), the illustrative values h = 0.85 m, H = 6 m, D = 52 m and X = 40 m give displacements of approximately 8.6 m across the road and 6.6 m along it under this full road-plane projection. These values describe the stated ideal geometry; they are not retrospective corrections to the case measurements. A one-dimensional mapping calibrated for a different feature locus or survey convention requires its own derivation. The feature height, calibrated plane, and coordinate convention must be established before an off-plane correction is applied. Without that information, its effect on the case-study estimates remains unresolved.

### 2.6. Frame Extraction and Feature Tracking

In the prospective workflow, frames should be decoded with FFmpeg or equivalent software while retaining presentation timestamps and the relationship between decoded frames and the source stream. A stable vehicle feature is tracked with software such as Tracker [33], and occluded observations are left absent. Gross-error screening should use documented rules, for example diagnostics from a preliminary fit [37], without silently removing inconvenient observations. Geometric corrections require the calibration evidence described in Section 2.3, Section 2.4 and Section 2.5. For this case, the supplied derived series tracks the rear edge of the car body and the motorcycle front wheel; it contains frame indices and image-abscissa values, but no presentation timestamps supplied for this analysis or quantitative Brown-correction residuals. The numerical analysis therefore uses the nominal timing and explicitly stated mapping assumptions; it does not demonstrate completion of every prospective acquisition or calibration step.

*Assignment of time to frames.* Verified presentation timestamps are preferred to ordinal frame numbers. Detected missing observations can be omitted without introducing a fictitious constant sampling interval, and duplicates must be assessed using both timing and image content. When timestamps are unavailable, the use of frame/15 must be stated explicitly and the estimates interpreted conditionally. A global frame-rate perturbation cannot represent all consequences of unobserved frame loss, duplication, or irregular sampling. Blurred or partially occluded observations are excluded or assigned an explicitly justified observation model; gaps are not filled with invented measurements.

### 2.7. Two-Phase Kinematic Model and Transition Estimation

For a vehicle observed first in approximately uniform motion and subsequently in approximately constant deceleration, a parsimonious description is a two-phase law continuous in position and velocity at the transition instant t*:

q(t) = q* + v_0_ (t − t*), t ≤ t*;   q(t) = q* + v_0_ (t − t*) + ½ a (t − t*)^2^, t > t*, (4)

where v_0_ is the pre-deceleration speed and a is the (negative) acceleration. For a given trial value of t*, the model is linear in the parameters β = (q*, v_0_, a) [38,39], so that with the design matrix A whose i-th row is [1, (t_i_ − t*), ½ (t_i_ − t*)^2^ 1(t_i_ > t*)] the least-squares estimate is:

(5) $$\hat{\beta }={\left(A^{T}A\right)}^{-1}A^{T}q,\;\mathrm{Cov}(\hat{\beta })={\hat{\sigma }}^{2}{\left(A^{T}A\right)}^{-1},\;{\hat{\sigma }}^{2}=\mathrm{RSS}/(n-p),$$

where RSS is the residual sum of squares and p = 3 is the number of linear coefficients conditional on a fixed t*. After estimating t* from the same observations, the residual noise scale uses RSS/(n − 4), a local regular-model approximation for the four fitted parameters. Equation (5) at a fixed transition gives conditional coefficient covariance, not covariance after estimating the transition. The estimator scans the admissible transition range, continuously refines every detected local minimum and the boundary-adjacent intervals, and compares both endpoints. The nominal 95% profile-F acceptance criterion [39] is RSS(t*) ≤ RSSmin [1 + F0.95; 1, n−4/(n − 4)]. Threshold roots define its interval components; disconnected sets and boundary truncation are retained. This nonlinear breakpoint approximation requires simulation-based coverage assessment [40]. Observations on both phases are needed for identification. For the car, q(0) = 0 expresses the selected tracked-feature reference, not independently measured physical contact. Global-polynomial derivatives are legitimate kinematic baselines, although a global polynomial may inadequately represent a change of regime. The covariance expression in Equation (5) assumes independent, equal-variance coordinate errors. For correlated errors with covariance Σq, the corresponding OLS coefficient covariance is (A^T^A)^−1^A^T^ΣqA(A^T^A)^−1^. In particular, subtraction of a noisy tracked-feature reference creates shared coordinate errors in the constrained late-car fit. Its analytical check uses that covariance, and the joint Monte Carlo calculation propagates the common reference explicitly; the independent-error expression is not applied to that fit.

### 2.8. Collision Instant and Position and Inter-Vehicle Distance

The reported first-contact frame supplies the temporal reference for the case calculation. At nominal 15 fps, uncertainty in selecting that frame is represented by discrete offsets of −1, 0, and +1 frame, separately from a global-rate scenario. A tracked body-feature position is distinct from the physical point of first contact; an unknown geometric offset cannot be treated as zero measured uncertainty. Distances computed from fitted feature trajectories are therefore conditional on the chosen reference conventions. The auxiliary recording was reported as supporting qualitative event alignment. Its reported offset of 82 frames is not a measured common-clock synchronisation, an independent timing check or evidence that sampling is uniform. The available computational data do not permit verification of overlay timestamps or drift, and no GPS-based synchronisation is assumed.

### 2.9. Stopping Distance and Conditional Stopping Scenarios

The following-vehicle stopping distance is approximated by:

S = v_0_ (t_1_ + t_2_ + ½ t_3_) + v_0_^2^/(2 a_b_), (6)

Here t_1_ is the reaction time, t_2_ the actuation delay, t_3_ the deceleration rise time, and a_b_ the positive magnitude of fully developed braking deceleration [1,2]. The critical time is obtained from the uniform branch of Equation (4), and the time at which the fitted inter-vehicle separation reaches a chosen perception distance is compared with it. This is a fixed-reference stopping scenario; it does not model evasive steering, a changing contact configuration, or all possible interactions. The triangular reaction-time distribution on [0.7, 1.5] s with mode 1.0 s is a scenario informed by the variability discussed in [41], rather than a measured driver-specific distribution. Likewise, a_b_ uniformly distributed on [6, 8] m/s^2^ is an assumed braking scenario. Reference [42] concerns engine braking without brake application and does not substantiate that service-braking interval. The resulting fraction P(λ_p_ > λ_cr_) is conditional on these choices and on extrapolation of the motorcycle motion before its first observation; it is not an empirically calibrated probability that the real collision was avoidable. Equation (6) represents deceleration build-up by the effective delay t_3_/2 while retaining v_0_ in the fully developed braking term. It is an engineering approximation, not the exact integral of an arbitrary braking-rise profile. This model-form approximation is not quantified by the stated input-propagation intervals.

Within each stopping-scenario draw, Xc is a fixed spatial target, although its value varies between draws with the selected feature reference. The critical time satisfies qB(−λ_cr_) = −S, so that on the extrapolated uniform branch λ_cr_ = (S + q*)/v_0_ − t*. The comparison time λ_p_ satisfies qA(−λ_p_) − qB(−λ_p_) = 100 m. The distance 100 m is an assumed perception threshold, not an observed driver perception distance. The actuation delay t_2_ = 0.20 s and rise time t_3_ = 0.50 s are held fixed; the specified reaction time and fully developed braking magnitude are varied. A shifted contact-frame draw changes the feature reference but does not turn this fixed-target scenario into a coupled interaction or evasive-manoeuvre model.

Terminology and interpretation. Throughout this article, speed denotes the scalar magnitude of motion, while velocity refers to a signed longitudinal component or a vector. The initial parameter v_0_ describes motion before the fitted onset of deceleration. It does not, by itself, prove brake application; slowing may include engine braking, rolling resistance or other mechanisms [42,43]. The speed immediately before contact is a different quantity. The positive fully developed braking-deceleration magnitude in the stopping scenario is distinguished from the negative fitted acceleration. Stopping-scenario fractions depend on the selected reaction-time, braking, and geometry assumptions; they are not unconditional probabilities of a historical event or a determination of driver responsibility.

### 2.10. Uncertainty Budget and Monte Carlo Propagation

The Monte Carlo calculation propagates specified input distributions through the fitted measurement model [22,23]. Each absolute road-coordinate observation receives one independent Gaussian perturbation before reference subtraction. The residual-derived standard deviations are 0.275 m for the early car line, 0.157 m for a free-intercept late car quadratic and 0.283 m for the fitted motorcycle law, using residual degrees of freedom n − 2, n − 3 and n − 4, respectively. These are assumed noise scales, not independently characterised localisation errors. The same noisy car observation defines Xc and is subtracted from both tracks, preserving the induced shared-reference covariance; no additional independent reference or coefficient-covariance draw is added. The controls receive independent assumed rectangular survey perturbations of ±0.20 m and image-abscissa perturbations of ±0.15 m. A rectangular global-rate perturbation of ±0.5% and equiprobable contact-frame shifts −1, 0 and +1 are assumed. For −1, car frame 167 is excluded from the pre-contact fit; for +1, Xc = 2 × 167 − X166 uses the same noisy absolute coordinates. The two-point projectivity is solved in every draw, preserving covariance between its coefficients. The car segments and the continuously profiled motorcycle transition are refitted. There are 20,000 joint draws, 4000 draws per one-at-a-time component and per separate scenario, and 4000 per isolated survey-range comparison. Reaction and braking inputs follow Section 2.9. Percentile intervals and boundary frequencies are reported. Unknown residual distortion, local timing irregularity, lateral or height bias and feature-to-contact offsets are not quantified by this budget; no hypothetical height scale is used as a case-specific bound.

Dependence and model completeness. Type A and Type B describe how uncertainty is evaluated, not whether errors are independent. Calibration parameters are shared across frames, and common survey errors can correlate the transformed positions. Manual tracking can also contain temporal dependence. A general linearised propagation uses u_c_^2^(y) = c^T^Σc, including off-diagonal covariance terms; summing only c_i_^2^u^2^(x_i_) is justified only when the corresponding covariances are negligible. Joint Monte Carlo sampling must preserve the assumed dependencies. The same observation error must not be counted again through both residual-based parameter uncertainty and an independently added tracking component. One-at-a-time perturbations are sensitivity diagnostics, and agreement of their summed variances with a joint calculation under independence does not demonstrate that the real errors are independent.

### 2.11. Identifiability of the Two-Phase Model and the Uncertainty of the Transition Instant

The transition search is performed in canonical frame-based time, then transformed consistently with each framerate and time-origin draw. For the nominal motorcycle observations, its admissible range is [−3.400, −0.467] s. A broad scan locates candidate minima; continuous refinement and threshold roots remove the earlier fixed-grid discretisation. Search limits remain computational choices, not a prior distribution or evidence about the transition. Conditional on t*, the model is linear in the other coefficients, so fixed-transition covariance and uncertainty after refitting t* describe different quantities. A narrow profile minimum or a nominal 95% acceptance interval does not establish correctness of the motion law. Flat or disconnected profiles, boundary estimates, and intervals reaching a search boundary must be reported; widths limited by the search window can understate the extent of an unbounded acceptance set.

The conditional uncertainty of v_0_ can be written in closed form for the uniform branch alone. For n equally spaced frames with interval Δt and residual standard deviation σ, the slope of a straight line has the standard uncertainty:

(7) $$u_{\mathrm{slope}}(v_{0})=\sigma /{\left[\Sigma_{i}{\left(t_{i}-\bar{t}\right)}^{2}\right]}^{1/2}=(\sigma /\Delta t)\;{\left[12/(n(n^{2}-1))\right]}^{1/2},$$

Equation (7) must use the actual observation times when frames are missing. Eight consecutive frames at 15 fps with σ = 0.28 m give a conditional slope standard uncertainty of approximately 0.65 m/s. Eleven consecutive frames give approximately 0.400 m/s (2.88 km/h expanded uncertainty at k = 2) and are the minimum integer count below 3 km/h under these assumptions; ten frames give about 3.33 km/h. The actual motorcycle early observations include frame 123 after a gap and are not eight equally spaced consecutive frames. Neither this slope calculation nor a covariance matrix at fixed t* includes uncertainty in estimating the transition or misspecifying the motion law. The comparison with full propagation in Section 3.2 is conditional on the same adopted geometry and nominal time base. These illustrative counts do not establish a universal acquisition requirement.

A transition inside an occlusion is inferred from the relationship imposed between the visible branches, not directly observed. Profile-interval coverage depends on that motion law, the observation pattern, and the noise model. For the motorcycle, its conditional nominal profile is reported with generating-model verification and additional mismatch tests. For the car, early residual curvature and historical deletion/timing sensitivity undermine interpretation of a two-phase transition as physical braking onset. Its early mean slope and late quadratic motion are therefore reported separately. A fitted transition can be an effective change time under gradual deceleration, and absence of an observed early branch can make pre-transition quantities unidentifiable.

### 2.12. Analytical Sensitivity Coefficients and Verification of the Monte Carlo Propagation

For a local linearisation, u_c_^2^(y) = c^T^Σc includes the covariance of the specified inputs; a sum of squared contributions applies only to inputs modelled as independent. The global rate has c_f_ = v_0_/f for speed. Perturbing a control changes both projective coefficients, the reference, and the fitted motion parameters. The complete control-point sensitivity is therefore obtained by differentiating the entire continuously refitted pipeline with respect to each surveyed or image control coordinate. The two derivatives are combined with their stated input covariance. A scale-only approximation based on control spacing omits perspective and refitting effects and is not the complete first-order calculation used in Section 3.2. For the stopping distance and critical time, the reaction, and braking derivatives are

∂S/∂t_1_ = v_0_,   ∂S/∂a_b_ = −v_0_^2^/(2a_b_^2^), ∂λ_cr_/∂t_1_ = 1, ∂λ_cr_/∂a_b_ = −v_0_/(2a_b_^2^), (8)

The contact-frame scenario changes the temporal origin and the tracked-feature reference together. Its standard time uncertainty is √(2/3)/15 = 0.0544 s for the three equiprobable offsets, rather than 1/(15√3) for a continuous rectangle. The analytical checks in Section 3.2 evaluate all three reference outcomes exactly. A pure time-origin shift leaves motorcycle speed and acceleration invariant; their remaining frame-only numerical dispersion is negligible. The moving reference still changes the critical time. Under the specified distributions, the reaction-time standard uncertainty is 0.165 s, and the braking-deceleration standard uncertainty is 0.577 m/s^2^. For the ideal full road-plane projection in Section 2.5, ∂(QX − X)/∂h = HX/(H − h)^2^. This derivative does not transfer unchanged to a different one-dimensional image-abscissa model, and no unresolved height or lateral term is represented as a quantified case contribution.

The analytical checks in Section 3.2 compare complete local first-order sensitivities, or the exact discrete reference calculation, with the corresponding one-at-a-time propagation. Agreement checks the numerical treatment of the stipulated inputs; it cannot establish their empirical distributions, independence, or completeness. The survey and image-control derivatives include the changing scale and perspective and the continuously re-estimated transition. Braking enters nonlinearly, so a local derivative and a finite-width rectangular propagation need not agree exactly. Likewise, agreement of an OAT quadrature sum with joint dispersion does not prove an exact variance allocation [44,45,46] or exclude unmodelled systematic errors.

### 2.13. Numerical Implementation, Reproducibility and Confidentiality of the Source Material

The corrected computation uses Python 3.12.14, NumPy 2.3.5 and SciPy 1.18.1, with Matplotlib 3.5.1 for figures. It reads the anonymised coordinates, preserves their observation mask, solves the exact stated projectivity, and fits the motion laws before propagating the specified inputs. The master seed is 20261019; the joint stream uses 20261020, and other seeds, input hashes, counts, and stage timings are retained in machine-readable output. The completed 20,000-draw run, including its OAT, synthetic, scenario, survey, and figure stages, took 681 s on the revision environment; the later figures-only rerun is a separate timing. Runtime and floating-point results are environment-dependent. Deterministic checks address reference reuse, time-origin invariance, rate scaling, the pre-contact mask, frame-66 evaluation, and agreement with a separately optimised nonlinear fit. They verify computational consistency, not source geometry, timing, or physical speed. Algorithm 1 summarises the implemented processing stages and their outputs.
**Algorithm 1.** Processing pipeline as implemented in the supplementary code (inputs: anonymised per-frame coordinates, control-point survey, nominal frame rate, collision frame).**Step****Operation****Output**1Use verified frame timestamps where available; here assign nominal frame/15. Apply the explicitly assumed exact projectivity and tracked-feature reference; case distortion correction is not verified.Time and coordinate inputs with provenance and limitations2Fit A early line and late quadratic separately. For B, scan the admissible transition range, continuously refine candidates, and locate profile-F roots; use n − 4 for fitted B residual variance.Retained kinematics, conditional covariance, profile components, and boundary diagnostics3Distinguish the tracked-feature reference from physical contact; compute separation, approximate stopping distance, and conditional critical/perception times; flag extrapolation.Xc, Tc, S, λ_cr_, λ_p_ and their interpretation4Sample each absolute tracking error once; reuse the same noisy reference for both tracks. Perturb controls, global rate, and contact frame; draw t_1_ and a_b_; refit (20,000 draws).Conditional dispersions, percentile intervals and scenario fraction P(λ_p_ > λ_cr_)5Propagate each component separately (4000 draws); compare complete first-order derivatives or exact discrete reference outcomes.OAT diagnostics and propagation checks (Section 3.2)6Verify the estimator with 2000 actual-mask synthetic trajectories; separately assess matched baselines, geometry, component rankings, observation designs, correlation, and mismatch.Bias, RMSE, interval coverage/width and boundary outcomes (Section 3.1 and Section 3.3; Tables S1–S6)7Retain input hashes, scripts, versions, seeds, counts, stage timings, and machine-readable outputs; generate figures and tables from the same final outputs.Reproducible conditional numerical record

Original recordings, individual frames, and identifying case materials are restricted and are not reproduced here. The derived numerical dataset consists of anonymised tracked-feature image abscissae, frame indices, and stated control correspondences. Together with the specified algorithms, seeds and machine-readable outputs, these inputs support reproduction of the conditional calculations. They do not supply missing presentation timestamps, independent calibration measurements, a surveyed physical contact point or an independent reference speed. Synthetic data and output tables concern explicitly stipulated experiments and carry no claim of recovering those missing case measurements.

### 2.14. Validation Design

The evidence is separated into distinct levels. Synthetic verification assesses an estimator under a known data-generating model; robustness requires scenarios that depart from the fitted assumptions. Agreement between numerical implementations using the same coordinates checks the estimation stage but not upstream tracking, calibration, or timing. Trace measurements and an auxiliary camera could provide additional case evidence if independently documented and checked; those source materials are not available in the supplied computational dataset and are not treated here as verified agreement. End-to-end validation requires an independent reference trajectory or speed, such as a characterised GNSS/RTK measurement [47], and is not established by this case. A controlled campaign should cover speed, deceleration, observation geometry, and occlusion. Non-collision manoeuvres can assess much of the measurement chain; a staged impact would address additional contact-identification questions under a specialised safety protocol. Such a campaign would require collaboration with a research and development organisation that operates controlled crash-test facilities, which are not currently available to the authors; no specific programme has been arranged, and future collaboration of this kind would be welcomed. Future controlled recordings should use a higher, independently verified frame rate, retain the complete frame timestamp sequence and include an independent motion reference. Increasing the sampling rate can improve temporal resolution, but cannot by itself correct inaccurate geometry, motion blur or undocumented frame timing.

## 3. Results

### 3.1. Synthetic Verification

The generating-model verification uses the 36 actual observed motorcycle frames from 113 through 163, including frame 123 and excluding missing frames 120–122 and 124–135. In 2000 independent trajectories, v_0_ = 30.0 m/s, a = −5.0 m/s^2^, t* = −2.80 s and Gaussian coordinate noise σ = 0.27 m are known. Table 2 reports the bias and dispersion of the continuously refined estimator. Profile-interval coverage is 95.5% (Monte Carlo standard error 0.46 percentage points); mean search-window interval width is 0.600 s. The fitted transition reaches a search boundary in 0.25% of runs, and the acceptance interval reaches a boundary in 10.7%; such widths are restricted by the search window. No disconnected intervals occurred. This verifies performance under the generating model, not under unknown distortion, timing error, correlated tracking, or another motion law. Those departures are examined separately in Section 3.3.

### 3.2. Application to an Anonymised Case

The case is a rear-end collision on a straight, dry, two-lane one-way carriageway with a speed limit of 60 km/h, in daylight, between a passenger car (vehicle A) travelling in the left lane and decelerating before an opening in the median, and a motorcycle (vehicle B) approaching from behind in the same lane. Two surveillance cameras at a nearby premises recorded the event (Table 1). All identifying information has been removed; no video frames are reproduced. The video files, the individual frames and any stills are confidential parts of the investigation file and are neither reproduced nor shared (Section 2.13); the analysis below rests entirely on the derived, anonymised measurements. The scene was surveyed during the forensic inspection; the original schematic places the reported event reference 11.9 m ahead of a survey reference line near the median opening (Figure 1a). That reported schematic position does not independently establish the physical contact point; the computation uses the tracked-feature reference defined in Section 2.2.

*Time base and calibration.* The 15-fps assignment remains nominal because primary-camera presentation timestamps were not available for this analysis. The ±0.5% global-rate distribution is an assumed scenario. The one-dimensional projectivity imposes q = 0 ↔ X = 0, hence b = 0, as an additional unverified correspondence; two other correspondences are q = −43.0 m to X = −43.9 m and q = 38.7 m to X = 37.8 m. Their exact solution, used consistently for nominal and perturbed calculations, is a = 0.99718766901 and c = 0.000540832883 m^−1^. The earlier rounded values 0.99719 and 0.00054 m^−1^ are retained only as historical inputs. The local derivative a/(cq + 1)^2^ is position-dependent; q is a metrically scaled image abscissa, so this derivative alone is not a metres-per-pixel scale. Independent check points, a documented origin correspondence, and quantitative residual-distortion data are unavailable.

**Figure 2:**
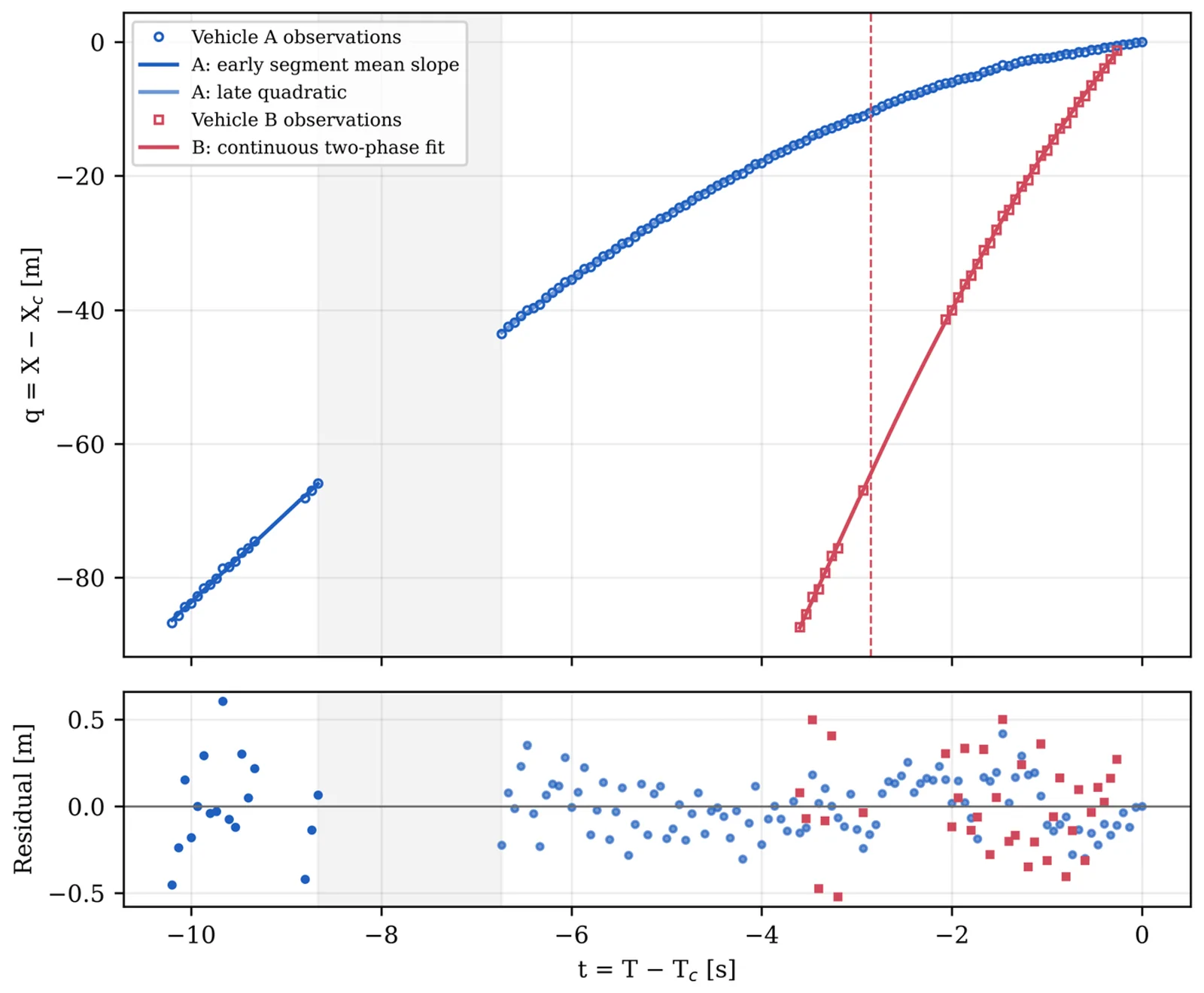
Observed positions, retained fits and residuals. Vehicle A uses a separate early line and late quadratic; vehicle B uses the continuously profiled two-phase law. The early car line estimates its segment mean slope. All fits remain conditional on the adopted geometry and nominal time base.

**Figure 3:**
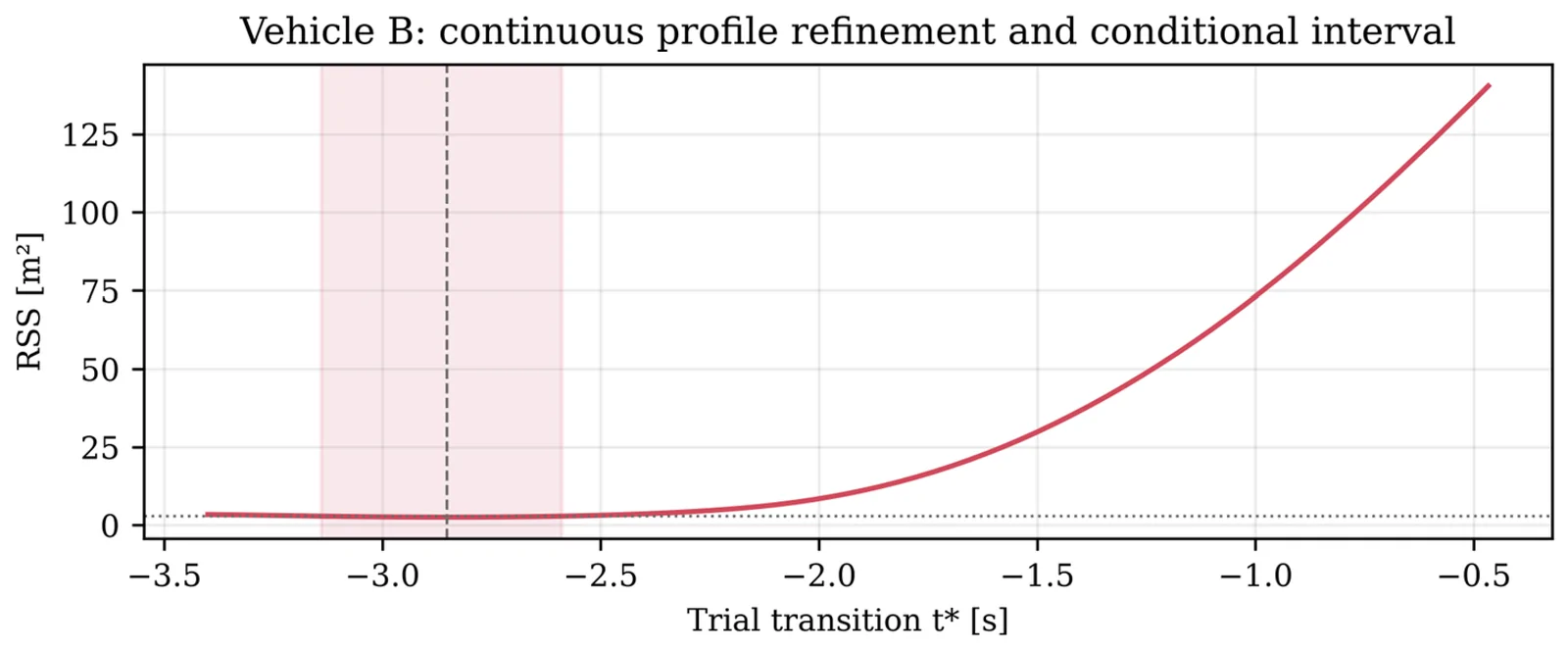
Motorcycle RSS profile with continuous minimum refinement and the conditional nominal 95% profile-F interval. The search window and interval describe the fitted two-phase model; they do not establish physical braking onset or model correctness.

*Tracking and fitting.* Vehicle A has 119 observations over frames 14–167, with missing frames 28–34 and 38–65. Vehicle B has 36 fitted observations over frames 113–163, with missing frames 120–122 and 124–135. Frame 167 defines the nominal reference time, 11.133 s at 15 fps. The motorcycle fit gives v_0_ = 30.895 m/s, a = −5.095 m/s^2^ and t* = −2.853 s; its profile interval is [−3.141, −2.589] s (Table 3). For the car, the 17 early observations give a mean slope of 13.305 m/s (47.9 km/h). A quadratic fit to that segment gives acceleration −1.31 ± 0.55 m/s^2^ as a conditional standard uncertainty, so a constant early phase is not established. The 102 nominal late observations are fitted separately with the shared tracked-feature reference q(0) = 0, giving a = −1.440 m/s^2^. The original, discretised car two-phase estimate changed from 12.537 to 12.219 m/s on deleting frames 14–21 and to 11.702 m/s on deleting frames 14–36 (changes −0.318 and −0.835 m/s). These explicit historical sensitivity checks correct the earlier claim of a change below 0.1 m/s; they are not new v2 estimates. A hypothetical early-segment time shift improves the original fit but does not establish duplicated frames. The original narrow car initial-speed interval is therefore not retained as a validated determination. Figure 2 presents the observed positions, retained motion fits and residuals; Figure 3 shows the continuously refined motorcycle transition profile and its conditional confidence set.

*Computational consistency.* The corrected estimates are calculated from the supplied anonymised CSV coordinates using exact control-point coefficients and continuous transition refinement. The original forensic report quoted MATLAB R2025b estimates of 12.542 m/s for the car and 30.854 m/s for the motorcycle. Agreement with historical estimates concerns shared coordinates and related estimation assumptions. The historical car estimate is not the retained early-segment mean slope, and numerical agreement does not resolve its model sensitivity or constitute independent physical validation.

**Figure 4:**
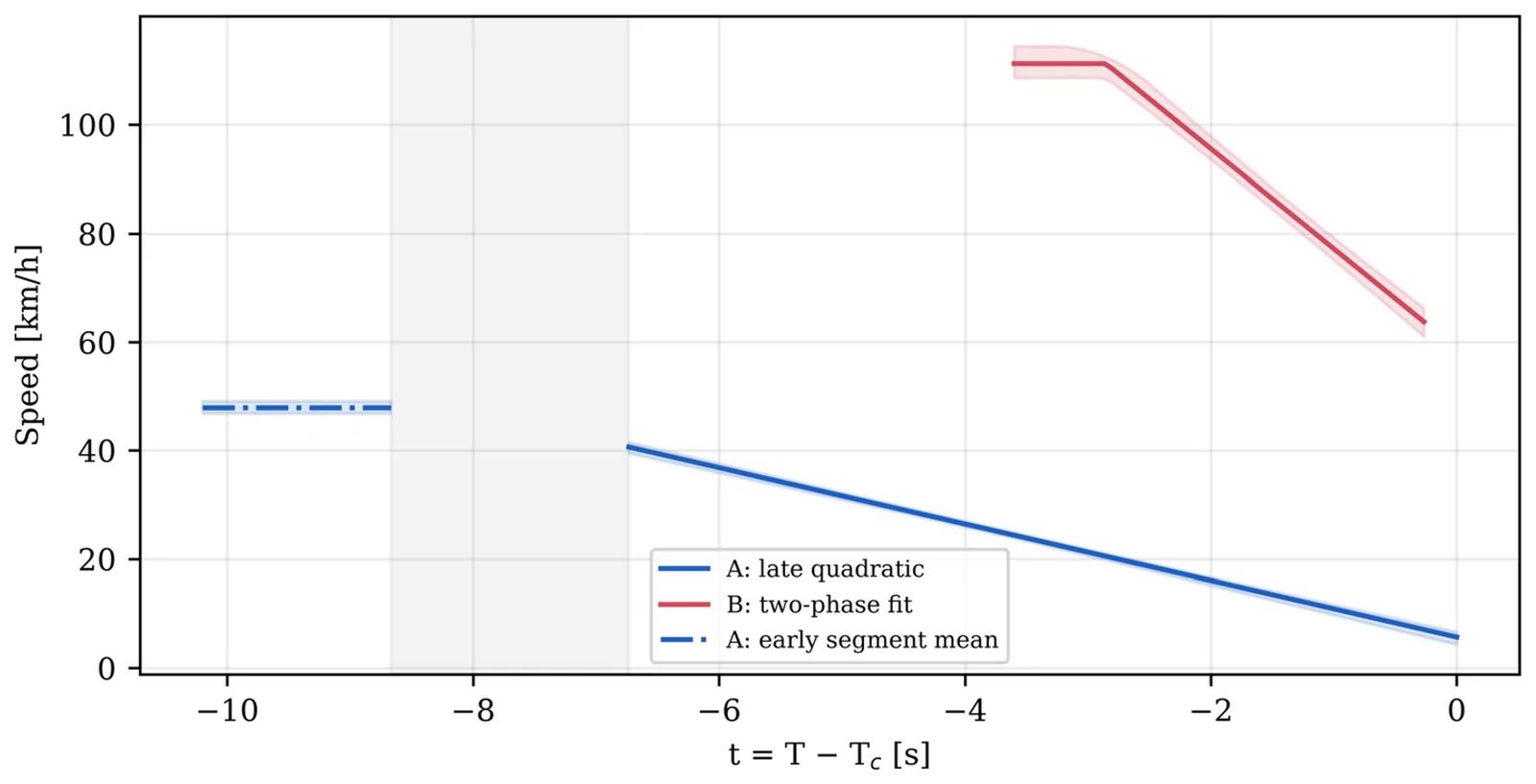
Fitted velocity summaries conditional on the specified inputs. Shading for the late car law and motorcycle law shows pointwise 2.5th–97.5th Monte Carlo percentiles, not simultaneous trajectory coverage. The early car segment shows its fitted mean slope with a separate 2.5th–97.5th Monte Carlo percentile band. It is not a verified constant-speed phase.

**Figure 5:**
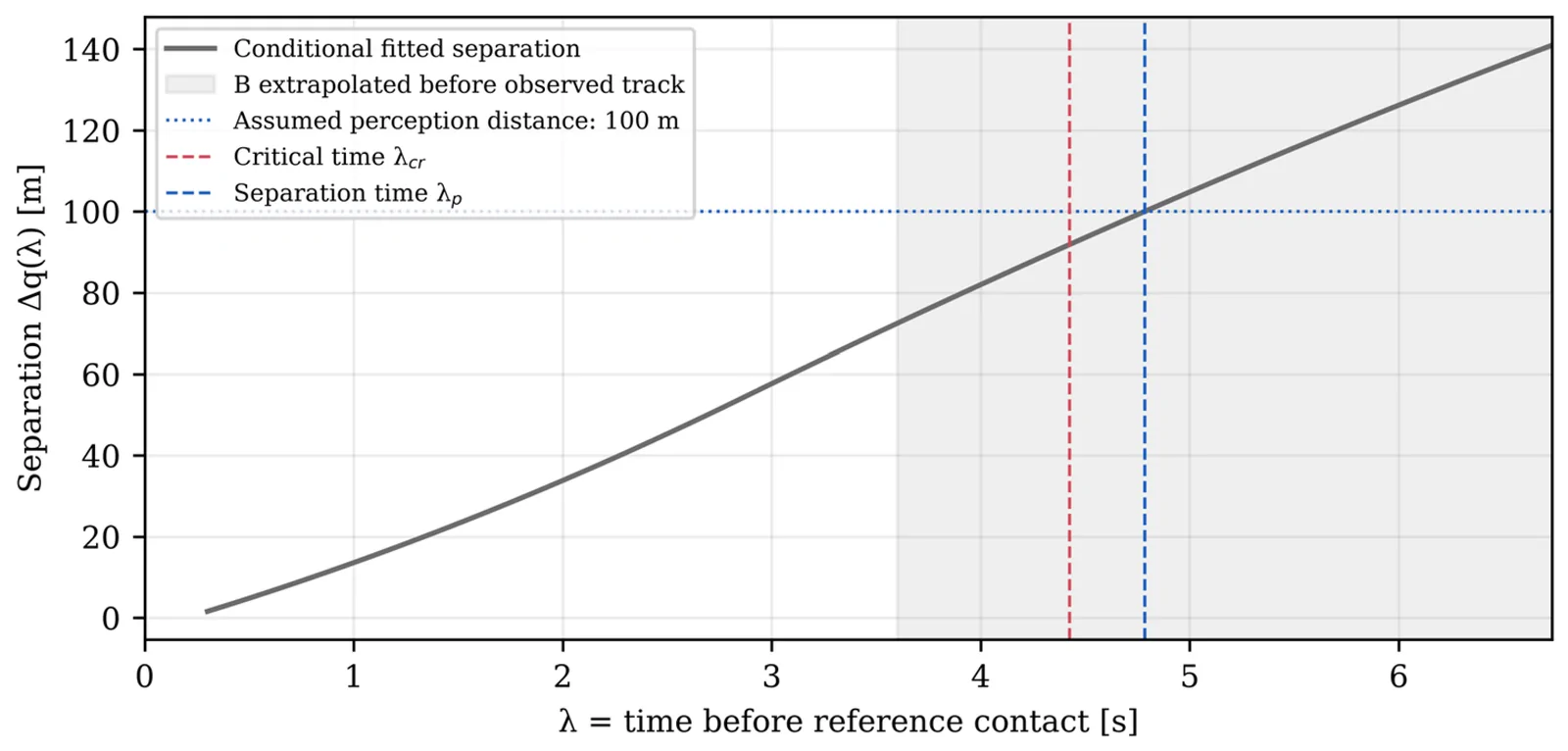
Conditional fitted separation and stopping-scenario times. The marked region requires backward extrapolation of the motorcycle trajectory beyond its first observation. The reference is the tracked car feature; the plot does not locate an independently measured physical contact point.

*Conditional stopping scenario.* Xc = 38.74 m is the mapped car feature at frame 167, not an independently surveyed physical contact point. The late car law gives 5.7 km/h at that reference time and 40.6 km/h at observed frame 66; the latter is evaluated at (66 − 167 − shift)/fps in every draw. The motorcycle law is not validated through its final fall. With t_1_ = 1.0 s, t_2_ = 0.2 s, t_3_ = 0.5 s and a_b_ = 7 m/s^2^, the approximate stopping distance is 113.0 m and λ_cr_ = 4.43 s. The fitted 100 m separation occurs at λ_p_ = 4.78 s. Both times precede the first motorcycle observation, 3.60 s before the reference, and require backward extrapolation of its uniform branch. The fraction satisfying λ_p_ > λ_cr_ is 0.837 under the stated distributions, with Monte Carlo standard error 0.003. This is simulation precision for a conditional scenario fraction, not a verified real-event probability. Table 4 and Table 5 report the propagated conditional uncertainty. Figure 4 shows the fitted velocity summaries and their conditional uncertainty displays; Figure 5 shows the separation curve, extrapolation region, and stopping-scenario times.

**The conditional distributions are summarised by Table 5; the associated numerical outputs retain the percentiles, seeds and boundary diagnostics.**

*Analytical checks.* The OAT quadrature sums are 0.413 m/s for motorcycle speed, 8.06 m for stopping distance, and 0.261 s for critical time, compared with joint dispersions of 0.419, 8.07, and 0.261, respectively. Table 6 uses complete survey and image-control derivatives through the refitted mapping. The survey speed derivatives with respect to the two road controls are (−0.9585, −0.2959) s^−1^. The exact three-point contact-reference calculation gives u(λ_cr_) = 0.0514 s while leaving motorcycle speed and stopping distance invariant. For late-car reference-time speed, covariance including the shared reference gives 0.0944 m/s, compared with tracking-only Monte Carlo 0.0953 m/s. These compatible checks assess propagation under the assumed model; their agreement does not establish empirical independence or budget completeness.

*Trace evidence and the auxiliary camera.* Original images, overlay timestamps, and independent trace measurements are absent from the supplied computational dataset. Accordingly, this analysis does not claim verified agreement with tyre or scrape traces, damage geometry, a second calibrated trajectory, or an independent overlay-based timing check. The reported 82-frame event alignment is retained as contextual information only; it neither establishes a common clock nor excludes drift. Quantitative lateral position or feature-to-contact offsets would require additional calibration and source evidence.

### 3.3. Additional Synthetic Comparisons

Matched estimands. We compared seven estimators on 500 common synthetic trajectories using the actual 36-observation motorcycle mask. Speed was evaluated at the prespecified midpoint of the first adjacent pair, t = −3.5667 s (truth 30 m/s), and acceleration at t = −1.0 s (truth −5 m/s^2^). Adjacent-frame differences, endpoint displacement, separate phase-wise OLS, global polynomials of degrees 2–4 and the continuously profiled two-phase law were compared against those same targets. Endpoint and phase-wise estimates receive true phase membership and are oracle benchmarks. The known Gaussian noise σ = 0.27 m is used for every speed/acceleration interval; the two-phase interval uses the joint local four-parameter Jacobian. Non-segmented methods do not estimate transition time. Tables S1–S2 specify the full bias, SD, RMSE, coverage, interval width, and simulation precision for these comparisons.

The two-phase speed RMSE is 0.450 m/s, compared with 0.484 m/s for the oracle early line and 5.855 m/s for adjacent-frame displacement. The two-phase speed interval covers 95.2% of runs, but acceleration coverage is 92.6% (MCSE 1.2 percentage points), below nominal. Its separate transition profile covers 94.0% (MCSE 1.1 percentage points), with mean search-window width 0.600 s; 0.4% of estimates and 11.6% of intervals reach a search boundary. Global-polynomial misspecification can produce narrow intervals with poor coverage. These outcomes describe one stipulated motion law and observation mask; they establish neither universal advantage nor real-case accuracy.

Amortised median per-track evaluation times are 0.0069 ms for the separate phase-wise regressions and 2.701 ms for the two-phase method. Reusable design setup, data reading, calibration, and noise generation are excluded. Linear methods apply cached estimators and interval weights; the two-phase workload additionally includes continuous profiling, joint-Jacobian covariance, and profile-F endpoints. Table S1 specifies interquartile times, information supplied, and the implementation scope. The recorded host is an Intel(R) Core(TM) i7-5500U CPU @ 2.40GHz (Intel Corporation, Santa Clara, CA, USA); these unequal workloads and environment-specific times are not a universal complexity comparison.

Component rankings. Seven conditions use 500 matched draws each to propagate tracking, survey-control, image-control, and global-rate errors separately and jointly through a synthetic forward/inverse projectivity. The origin correspondence is exact by construction; survey and image half-widths are set to ±0.20 and ±0.15 m, and the rate half-width to ±0.5%. Gaussian image-coordinate noise is scaled to the local road-equivalent tracking standard deviation. Conditions vary in sampling, early observations, tracking noise, and deceleration (Table S3). At local road-equivalent σ = 0.27 m, tracking contributes 87.4% of normalized OAT speed variance and joint u(v_0_) is 0.502 m/s. At σ = 0.03 m, survey becomes the largest component (46.1%), tracking contributes 6.5%, and joint u(v_0_) is 0.191 m/s. These shares are conditional diagnostics, not exact nonlinear variance allocations or measured case contributions. The designs include extrapolation outside the control interval; boundary frequencies are retained.

Geometry sensitivity. A separate road-plane experiment specifies a pinhole camera at (0, −35, 10) m aimed at the origin, a 1920 × 1080 pixel image with a focal length of 1200 px, a Brown forward radial coefficient k_1_, and a known constant-speed trajectory. Across 23 conditions, 500 realisations each vary 4, 8, or 12 non-collinear controls, distributed or clustered layouts, k_1_ = −0.15, 0 or +0.15, correction with the true coefficient, and control measurement noise. Image-to-road homographies use normalised DLT. Independent test points are excluded from calibration; the same noisy calibration maps every track and test point in a draw. The baseline control and track noise standard deviations are 0.5 and 0.25 px per coordinate; separate survey-noise scenarios use Gaussian standard deviations, distinct from case rectangular half-widths. Every feature is on the road plane, so the study cannot resolve elevated-feature geometry. Full design and error results are specified in Table S4.

With zero distortion and exact road controls, four distributed noisy image controls give a calibration residual near zero but independent-position RMSE 0.075 m (MCSE 0.002 m). With 12 distributed controls, the RMSE is 0.030 m; clustering them increases it to 0.267 m in this test domain. For 12 distributed controls and uncorrected k_1_ = +0.15, speed RMSE is 0.292 m/s, compared with 0.007 m/s when the true coefficient is supplied. Small determining-control residuals therefore do not establish independent geometric accuracy. Known-coefficient correction serves as an oracle reference, and these synthetic values do not estimate the unknown distortion in the case or survey accuracy.

Observation-design sensitivity. Continuous-profile experiments use 500 realisations per condition, varying missing-slot gap length (0, 12, 24), position (−8, 0, +8 nominal frames), early observation count (4, 8, 16), coordinate noise (0.10, 0.27, 0.50 m), sampling rate (10, 15, 30 fps) and deceleration (−3, −5, −7 m/s^2^), one factor at a time (Table S5). Approximately matched sampling-rate spans give speed RMSE values of 0.716, 0.615, and 0.416 m/s. The shift = −8 design has no observations before true t* = −2.8 s, and its search starts at −2.4 s. Its zero coverage is structurally inevitable; speed RMSE is 2.217 m/s, and 63.2% of boundary estimates describe that identification/search-domain failure. Four early observations give 97.8% coverage but 37.8% boundary estimates and 96.0% boundary-reaching intervals. Coverage alone is therefore insufficient; search-restricted widths and boundary frequencies accompany every condition.

Correlation and model mismatch. In 1000 realisations for each assumed AR(1) coefficient ρ = 0, 0.3, 0.6, and 0.8, the corresponding transition-profile coverages are 95.6%, 90.3%, 78.5%, and 69.1%. Noise has marginal σ = 0.27 m and is generated on the full frame sequence before masking. With a 0.6 s deceleration rise, the fitted transition bias relative to the physical onset is 0.292 s; onset coverage is 54.7%, while midpoint coverage is 94.8%. Table S6 specifies all correlation, ramp, and initially nonuniform-motion outcomes. The fitted transition can represent an effective change time under a mismatched law; neither continuous optimisation nor a nominal 95% profile threshold establishes a physical onset or repairs model mismatch.

## 4. Discussion

*Comparison with published methods.* Conditional uncertainty from a propagated model cannot be ranked directly against errors measured using an independent GPS reference. The present case has no independent speed ground truth, so its small numerical residuals do not demonstrate superiority over the methods reviewed in the Introduction. Baseline comparisons must use common synthetic observations with known truth and distinguish the quantity estimated by each method. A line fitted to an independently identified uniform segment estimates its slope; a global polynomial estimates time-varying derivatives; the two-phase fit estimates a model-specific initial speed and transition. The initial phase must not be selected with unreported knowledge of the true transition. CMTT [48] predicts surrounding trajectories using convolutional features of dynamic occupancy-grid maps and a transformer; MaTVT [49] models future multi-agent motion using historical trajectories, map context, and agent interactions. Those prediction tasks differ from retrospective estimation of motion from a short, partially observed forensic track. Their advantages motivate investigation of richer motion laws, but the present data do not support a fair trained-network benchmark or a claim that the two-phase model outperforms them.

*Sensitivity.* Tracking and transition refitting strongly contribute to motorcycle speed dispersion under the principal case assumptions, whereas reaction and braking assumptions drive most of the stopping-distance dispersion. The motorcycle estimate reaches a search boundary in 0.070% of joint draws. This ranking depends on the adopted noise scales, observation pattern, and geometry; Section 3.3 shows it changes under other synthetic conditions. In matched survey-only calculations with 4000 draws each, assumed rectangular half-widths ±0.05, ±0.20 and ±0.50 m give u(v_0_) = 0.029, 0.116 and 0.291 m/s, respectively. The wider comparison is a separate sensitivity, not a replacement for joint uncertainty or a measured survey bound. None of these allowances identifies the actual survey distribution. Unknown feature height, lateral geometry, residual distortion, and feature-to-contact offset remain unresolved; a small fitting residual cannot bound them.

*Applicability conditions.* Requirements depend on the actual observation span, visible phases, geometry, noise, and timing. Table 7 lists checks and illustrative design considerations rather than a guaranteed accuracy envelope. More frames can improve conditional precision but cannot repair unverified timestamps, systematic distortion, unknown feature height, or motion-model mismatch. A moving camera requires pose estimation and a time-varying mapping. Rolling shutter [50], strong compression, adverse visibility, and the final three-dimensional motorcycle fall remain outside the quantitatively validated scope of this case and the specified synthetic tests.

*Limitations.* The case study concerns one event and one camera geometry. No independent reference speed is available, so internal consistency checks cannot establish end-to-end accuracy. Synthetic tests quantify performance only for their specified trajectories, sampling patterns, and error models. Agreement with another implementation using the same coordinates does not independently validate tracking or calibration. The one-dimensional transformation uses two control points and an origin convention; additional surveyed controls and independent checkpoints are needed to assess geometric residuals and extrapolation. This analysis did not have individual presentation timestamps, leaving local timing errors unverified rather than demonstrably bounded. Feature height and the calibration plane also require reconciliation before a complete geometric uncertainty can be claimed. The final motorcycle motion includes yaw, lean, and falling, outside the planar model; any extrapolation to contact depends on additional assumptions. Manual tracking introduces operator dependence, and residual scatter does not by itself quantify systematic tracking bias. Automation may improve repeatability but requires validation. The reported auxiliary-camera event alignment has not been verified here against an independently calibrated trajectory or clock. These limitations constrain interpretation of the numerical uncertainty budget and prevent generalisation of the case estimates to other acquisition conditions.

Environmental and camera-motion limits. The reported case concerns daylight and a dry road; no adverse-weather validation is established here. Fog, snow, rain, reflections, and compression can change contrast, visibility, and feature localisation, while weather can separately alter the braking assumptions. Their quantitative effects require dedicated measurements or explicitly labelled synthetic image-degradation studies. A moving or PTZ camera violates the fixed mapping unless camera pose and, where relevant, focal length are estimated over time. Such an extension requires additional observations or calibration and has not been validated by the stationary-camera case. Outputs from this workflow may supply initial conditions and uncertainty ranges for PC-Crash or V-SIM, but agreement with a simulation driven by those same inputs is not an independent validation. No new commercial-software comparison is claimed.

## 5. Conclusions

The workflow makes the assumptions connecting image observations to kinematic and stopping quantities explicit and reproducible. The case study supports conditional numerical estimates, but missing timestamp and calibration evidence prevents a general accuracy claim. The motorcycle two-phase estimate remains reproducible; the car requires separate reporting of its early mean slope and late motion because a constant initial phase is not established. Synthetic verification with the actual observation mask and sensitivity analysis complement, but do not replace, an independent reference experiment. The stopping calculation is an explicitly conditional scenario involving extrapolation and assumed human-response and braking inputs. The practical outcome is a reproducible distinction between quantities supported by the available track, quantities dependent on a selected model, and uncertainties that additional measurements must resolve.

## Figures and Tables

**Table 1:** Recording and sensor parameters of the two cameras (anonymised).

Parameter	Camera 1 (Primary)	Camera 2 (Auxiliary)
Resolution	1920 × 1080 px	1920 × 1080 px
Nominal frame rate	15 fps	15 fps
Decoded frames/nominal duration	16,124/18 min (≈14.9 fps)	15,209/18 min (≈14.1 fps)
Timestamp regularity	PTS unavailable; assumed ±0.5% global-rate scenario	Reported irregular timing; source intervals unavailable for verification
Observation geometry	Reported lateral offset ≈ 52 m and height ≈ 6 m; full optical pose unverified	Reported oblique view; quantitative calibration unavailable
Use	quantitative (kinematics)	Reported qualitative event alignment only

**Table 2:** Synthetic verification of the continuously refined estimator using the actual motorcycle observation mask (2000 realisations; known Gaussian noise σ = 0.27 m).

Parameter	True Value	Bias	Standard Deviation
v_0_ [m/s]	30.00	0.035	0.435
a [m/s^2^]	−5.00	−0.035	0.229
t* [s]	−2.80	−0.002	0.147

**Table 3:** **Distinct** kinematic quantities for the two vehicles. The early car mean slope is not a proven constant initial speed. Fixed-transition least-squares uncertainty and jointly propagated uncertainty are distinguished; all are conditional on the stated model and inputs.

Quantity	Vehicle A (Car)	Vehicle B (Motorcycle)
Frames used/n	early 14–37/17; late 66–167/102 nominal	113–163/36
t* [s] (profile interval)	Not established	−2.853 (−3.141; −2.589)
Early mean slope/initial v_0_ [m/s] ([km/h])	13.31 (47.9)	30.90 (111.2)
Conditional LS u(speed) [m/s]	0.144	0.111 (fixed t*)
Joint MC u(speed) [m/s]	0.161	0.419
Conditional U(speed), k = 2 [km/h]	1.2	3.0
Late acceleration [m/s^2^]	−1.440	−5.095
Joint MC u(a) [m/s^2^]	0.036	0.196
Assumed absolute tracking σ [m]	early 0.275; late 0.157	0.283
Speed at reference time/frame 163 [km/h]	5.7	63.8

**Table 4:** One-at-a-time conditional standard uncertainties for motorcycle initial speed, approximate stopping distance, and critical time (4000 draws per component); joint propagation uses 20,000 draws. Entries below displayed precision are rounded to zero, including the frame-only speed artefact removed by continuous refinement.

Component	Type	Distribution/Limits	u(v_0_) [m/s]	u(S) [m]	u(λ_cr_) [s]
Absolute tracking and transition	A	Assumed Gaussian; shared reference; t* refitted	0.375	2.20	0.058
Control-point survey	B	Assumed rectangular ±0.20 m	0.116	0.68	0.017
Image-abscissa controls	B	Assumed rectangular ±0.15 m	0.091	0.53	0.013
Global frame rate	B	Assumed rectangular ±0.5%	0.090	0.53	0.004
Contact-frame reference	B	Discrete −1, 0, +1; moving reference	0.000	0.00	0.051
Reaction time	B	Assumed triangular 0.7/1.0/1.5 s	0.000	5.14	0.166
Braking deceleration	B	Assumed rectangular 6–8 m/s^2^	0.000	5.72	0.185
Joint simulation		20,000 draws	0.419	8.07	0.261

**Table 5:** Conditional Monte Carlo means, standard deviations and 95% percentile intervals (20,000 draws). The frame-66 quantity uses that observed frame’s time in each draw. Unknown systematic effects are excluded.

Quantity	Mean	u	95% Interval
A early mean slope [km/h]	47.9	0.6	46.8–49.0
B initial speed [km/h]	111.3	1.5	108.6–114.5
A late acceleration [m/s^2^]	−1.439	0.036	−1.512–−1.364
B acceleration [m/s^2^]	−5.11	0.20	−5.52–−4.74
A reference-time speed [km/h]	5.71	0.58	4.32–6.68
A speed at observed frame 66 [km/h]	40.60	0.38	39.81–41.37
B transition [s]	−2.86	0.13	−3.12–−2.60
Tracked-feature reference Xc [m]	38.74	0.30	38.20–39.39
Approximate stopping distance [m]	115.7	8.1	101.0–131.9
Critical time [s]	4.51	0.26	4.03–5.03
100 m separation time [s]	4.783	0.063	4.670–4.897
Conditional scenario fraction	0.837	MC SE 0.003	Not a physical confidence interval

**Table 6:** Complete local first-order checks and the exact three-outcome contact-reference calculation versus one-at-a-time Monte Carlo propagation. Each output triple is u(v_0_) [m/s]; u(S) [m]; u(λ_cr_) [s]. The controls include both projective coefficients and motion refitting.

Component	Sensitivity Calculation	Input Standard Uncertainty	First-Order/Exact u(v_0_; S; λ_cr_)	MC u(v_0_; S; λ_cr_)
Global rate	Complete time scaling and refit	Relative 0.5%/√3	0.0892; 0.5230; 0.0042	0.0901; 0.5282; 0.0042
Survey controls	Complete two-control Jacobian	0.20/√3 m each	0.1158; 0.6792; 0.0166	0.1164; 0.6823; 0.0167
Image-abscissa controls	Complete two-control Jacobian	0.15/√3 m each	0.0902; 0.5287; 0.0127	0.0910; 0.5333; 0.0128
Contact reference	Exact three outcomes; reference moves	√(2/3)/15 s	0.0000; 0.0000; 0.0514	0.0000; 0.0000; 0.0513
Reaction time	∂S/∂t_1_ = v_0_; ∂λ_cr_/∂t_1_ = 1	0.1650 s	0.0000; 5.0974; 0.1650	0.0000; 5.1403; 0.1664
Braking deceleration	∂S/∂a_b_ = −v_0_^2^/(2a_b_^2^)	0.5774 m/s^2^	0.0000; 5.6233; 0.1820	0.0000; 5.7182; 0.1851

**Table 7:** Application checks and illustrative design considerations; these are not guaranteed thresholds for U(v_0_) ≤ 3 km/h.

Condition	Check	Interpretation
Early visible phase	Use actual times and residuals	11 consecutive frames meet the illustrative slope-only target; no general guarantee
Decelerating phase	Assess curvature and visible span	Frame count alone does not establish identifiability
Spatial resolution	Report local pixel footprint	Convert localisation error with measured coordinate geometry
Controls and origin	Document correspondences and independent check points	Four non-collinear controls determine a plane homography; redundant controls and checks assess its adequacy
Viewing geometry	Measure pose and test extrapolation	No validated ±30° accuracy envelope
Tracked feature	Document height and physical contact offset	Road-plane and body-feature mappings differ; unresolved offsets are not bounded by residuals
Time base	Inspect frame PTS, gaps and duplicates	Nominal fps and global-rate scenarios do not verify local timing

## Data Availability

The derived anonymised coordinates, numerical inputs, code and results are supplied in the accompanying numerical material identified above. Original recordings, individual frames and still images are restricted because they form part of a confidential forensic examination. The supplied material supports reproduction of the conditional analysis; it does not supply a complete primary-camera timestamp sequence, independently checked geometric calibration, a physical feature-to-contact offset, or independent validation measurements. No public repository deposit is claimed.

## Supplementary Materials

The following supporting information can be downloaded at: https://www.mdpi.com/article/10.3390/s26216868/s1, Table S1: Matched synthetic speed estimates and computation times; Table S2: Matched synthetic acceleration and transition estimates; Table S3: Seven-condition synthetic component budgets; Table S4: Synthetic road-plane geometry sensitivity in 23 conditions; Table S5: Continuous-profile observation-design sensitivity; Table S6: Correlated tracking and motion-model mismatch (file Supplementary_Tables_S1-S6.xlsx); Sensors_Reproducibility_v2.zip: anonymised per-frame coordinates, stated control inputs, the corrected analysis and verification scripts and machine-readable results. The archive documents dependencies, seeds, commands, assumptions, and file hashes. Original recordings, still images and identifying case materials are excluded.

## Appendix A. Application Protocol

A1. Obtain the original recordings and inspect their metadata and frame presentation timestamps; distinguish measured intervals from nominal frame-rate assignments and document missing evidence. A2. Survey camera position, road geometry, and distributed control points, recording their uncertainties and reserving independent check points. A3. Estimate lens distortion and report coefficients and residuals. A4. Select the geometric mapping appropriate to the tracked feature and its height; assess calibration residuals and extrapolation. A5. Track stable features using their actual observation times, retaining occlusion gaps, and recording the selection rules. A6. Compare plausible motion laws, inspect residuals and observation-window sensitivity, and retain a two-phase fit only when supported; otherwise report separate observed segments or model-dependent scenarios. Report transition profiles, boundary solutions, and the conditions of interval coverage. A7. Identify the contact-time reference and distinguish tracked-feature coordinates from physical contact points, documenting unknown offsets. A8. Compute stopping-related quantities only as scenarios with explicit reaction, braking, timing, and geometry assumptions; mark extrapolation beyond each vehicle observation interval. A9. Propagate stated inputs jointly, including assumed dependence where relevant; distinguish conditional uncertainty from unresolved systematic effects and compare compatible first-order diagnostics. A10. Use independent reference measurements for physical validation when available; otherwise, label consistency checks appropriately. Archive anonymised observations, code, seeds, software versions and machine-readable results, excluding confidential recordings and frames. These steps are a prospective protocol; missing case evidence is not implied to have been collected.
